# Recombinant myostatin reduces highly expressed microRNAs in differentiating C2C12 cells

**DOI:** 10.1016/j.bbrep.2017.01.003

**Published:** 2017-01-17

**Authors:** Zachary A. Graham, Rita De Gasperi, William A. Bauman, Christopher P. Cardozo

**Affiliations:** a National Center for the Medical Consequences of Spinal Cord Injury, Bronx, NY, USA; b Medical Service, James J. Peters VAMC, Bronx, NY, USA; c Departments of Medicine, Icahn School of Medicine at Mt. Sinai, NY, USA; d Departments of Psychiatry, Icahn School of Medicine at Mt. Sinai, NY, USA; e Departments of Rehabilitation Medicine, Icahn School of Medicine at Mt. Sinai, NY, USA; f Departments of Pharmacologic Science and the Icahn School of Medicine at Mt. Sinai, NY, USA; g Friedman Brain Institute, Icahn School of Medicine at Mt. Sinai, NY, USA

**Keywords:** Myostatin, MiRNA, MyomiR, Activin receptor-like kinase, Myogenic differentiation, Atrophy

## Abstract

Myostatin is small glycopeptide that is produced and secreted by skeletal muscle. It is a potent negative regulator of muscle growth that has been associated with conditions of frailty. In C2C12 cells, myostatin limits cell differentiation. Myostatin acts through activin receptor IIB, activin receptor-like kinase (ALK) and Smad transcription factors. microRNAs (miRNA) are short, 22 base pair nucleotides that bind to the 3′ UTR of target mRNA to repress translation or reduce mRNA stability. In the present study, expression in differentiating C2C12 cells of the myomiRs miR-1 and 133a were down-regulated following treatment with 1 µg of recombinant myostatin at 1 d post-induction of differentiation while all myomiRs (miR-1, 133a/b and 206) were upregulated by SB431542, a potent ALK4/5/7 inhibitor which reduces Smad2 signaling, at 1 d and all, with the exception of miR-206, were upregulated by SB431542 at 3 d. The expression of the muscle-enriched miR-486 was greater following treatment with SB431542 but not altered by myostatin. Other highly expressed miRNAs in skeletal muscle, miR-23a/b and 145, were altered only at 1 d post-induction of differentiation. miR-27b responded differently to treatments at 1 d, where it was upregulated, as compared to 3 d, where it was downregulated. Neither myostatin nor SB431542 altered cell size or cell morphology. The data indicate that myostatin represses myomiR expression in differentiating C2C12 cells and that inhibition of Smad signaling with SB431542 can result in large changes in highly expressed miRNAs in differentiating myoblasts.

## Highlights

• Myostatin down-regulates the myomiRs in myoblasts prior to and during myotube formation.
• Inhibition of ALK signaling increases expression of highly-expressed miRNA in myoblasts.
• Myostatin and ALK inhibition has a greater effect on the miRNA profile at 1 D post-induction of differentiation.

## 1 Introduction

Myostatin (GDF8) is a small, 12 kDa peptide member of the transforming growth factor-β (TGFβ) superfamily that is secreted by skeletal muscle and is a potent negative regulator of muscle growth [1]. A knockout of myostatin leads to a ‘double-muscling’ phenotype in mice [2] and mimics the effects on muscle mass of naturally occurring mutations of the myostatin gene in cattle [3] and in a human [4]. Myostatin prevents muscle growth by suppressing hypertrophic signaling pathways and increasing transcription of genes that degrade skeletal muscle protein. [5] Myostatin signals through phosphorylation of Smad2 and Smad3 transcription factors. [6] Myostatin binds the membrane-bound activin receptor IIb (ACRIIB), a step necessary for activation of activin receptor-like kinases (ALK) 4 or 5, which then phosphorylate Smad2 and 3. In the cytosol, phosphorylated Smad2/3 inhibit Akt, a major kinase that responds positively to exercise and growth factors, such as IGF-1, to increase protein synthesis and inhibit protein breakdown [7]. Additionally, after phosphorylation by ALK 4 or 5, the Smad2/3 complex can bind Smad4 and translocate to the nucleus to increase transcription of genes, and partially activate major factors involved in muscle atrophy programs such as muscle atrophy Fbox (MAFbx) as seen with luciferase reporter studies [6].

Due to the deleterious effects of myostatin on muscle, it is not surprising that it is associated with conditions of frailty and muscle wasting [8], [9], [10]. One of the most severe forms of muscle wasting occurs following spinal cord injury (SCI). SCI leads to rapid decreases in muscle mass below the area of injury [11], [12], [13] and diminished hypertrophic signaling [14]. Previous work from our lab has shown that even though myostatin mRNA is not affected by SCI at 56 d post-injury in the paralyzed rat gastrocnemius, SCI still results in greater Smad2/3 nuclear protein localization and ACRIIB mRNA expression, suggesting greater myostatin signaling activation [12]. Additionally, we have found that SCI leads to dysregulation of highly expressed microRNA (miRNA) in paralyzed rat gastrocnemius muscle [15]. miRNA are ~22 base pair nucleotides that bind to the 3′-untranslated region (UTR) of target mRNAs. They are transcribed by RNA polymerase II into primary transcripts (*pri*-miRNA) and processed by the ribonuclease Drosha into pre-miRNA. Pre-miRNAs are then exported out of the nucleus by exportin-5 and further processed into an active form by Dicer [16]. Some miRNAs are expressed by miRNA genes while others are released from introns after mRNA splicing. Regardless of their origin, in almost all cases miRNAs repress translation of their target mRNA. miR-1, miR-133a/b and miR-206 are highly-expressed in skeletal muscle where they aid in differentiation of myogenic precursors and muscle function and are referred to as myomiRs [17].

Knowledge of the role of myostatin in the regulation of miRNAs in skeletal muscle development and homeostasis remains limited. Transcriptome analysis using deep sequencing supports a role for myostatin in regulating miRNA expression. Inhibition of myostatin in a transgenic mouse model overexpressing the myostatin propeptide revealed 57 differently expressed miRNAs as compared to wild-type littermates [18]. In a recent report it was shown that myostatin limits cell differentiation of C2C12 myoblasts [19]. Additionally, C2C12 cells aged by repeated population doubling have increased levels of myostatin gene expression and blunted rates of differentiation compared to parent lines at both 3 and 7 d after transition to differentiation media [20], [21].

We have observed that SCI is associated with decreased expression of miR-23a/b, miR-27b, miR-145 and miR-206; a pathways analysis implicated these miRNAs as being known or predicted to repress translation of multiple proteins involved in TGFβ family signaling [15]. These findings raised the question as to whether activation of Smad2/3 signaling in muscle might be responsible for some or all of the changes in miRNA expression observed in muscle after SCI. Little is known regarding the role of myostatin or Smad signaling in modulating expression of these miRNA. There is, however, evidence that these miRNA have a role in muscle homeostasis. miR-23a/b have been predicted to target E3 ligases in skeletal muscle and overexpression of miR-23b can limit muscle atrophy [22]. miR-27b can target myostatin mRNA and has been associated with fiber type transformation [23] while short hairpin induced inhibition of miR-206 resulted in myotube growth in a dose-dependent manner [24]. miR-486 is an intriguing miRNA in that it is highly expressed in skeletal muscle and targets FoxO1, a major growth-restricting transcription factor in skeletal muscle [25]. Moreover, Smad2/3 downregulates the Akt pathway, which otherwise reduces FoxO family activity [5]. Exogenous myostatin can inhibit Akt phosphorylation and decrease myotube size through Smad2/3 activation in culture [26] while constitutively active Akt reduces expression of the ACRIIB and can protect muscle cross sectional area [6].

Thus, there is ample evidence that miRNAs expressed in skeletal muscle regulate the expression of multiple key proteins in myostatin/ACRRIIB/Smad2/3 signaling as well as other signaling pathways that govern muscle protein catabolism and muscle atrophy and hypertrophy programs. The objective of the present study was to determine whether myostatin could regulate the expression of muscle-specific and highly expressed miRNAs that were identified as being differentially regulated in gastrocnemius muscle after SCI [15]. To achieve this objective, we evaluated the effects of myostatin and/or the ALK4/5/7 inhibitor SB431542 on miRNA expression in differentiating mouse C2C12 myoblasts, which are widely employed in studies of the intracellular signals responsible for muscle atrophy and hypertrophy as well as myogenic differentiation. In order to understand how these treatments affected cell hypertrophy and differentiation, cell size and expression of MyoD, a myogenic transcription factor, were determined.

## 2 Material and methods

### 2.1 Cell Culture

Mouse C2C12 myoblasts were grown to confluency in 6-well plates in proliferating media consisting of Dulbecco’s Modified Eagle’s Medium (DMEM) with 10% fetal bovine serum (FBS) and 1% penicillin/streptomycin (P/S). At confluency, the media was removed and cells were washed twice in 1x phosphate buffered saline (PBS) and covered with differentiating media consisting of DMEM supplemented with 2% horse serum and 1% P/S. Cells were harvested at 1 day (1 D) and 3 days (3 D) post-induction of differentiation. One day prior to cell harvest, media was supplemented with vehicle (4 mM HCl with 0.1% BSA), recombinant myostatin (R&D Laboratories) at 100 ng/ml and 1 µg/ml without or without co-incubation with 1 µM SB431542 (Sigma, St. Louis, MO USA) and 1 µM SB431542 alone. SB431542 is a small molecule that selectively inhibits ALK 4, 5 and 7 [27]; at 1 µM SB431542 has been shown to significantly reduce Smad2 phosphorylation in C2C12 cells [19].

### 2.2 Cell Morphology

Morphology of C2C12 cells was determined after seeding cells on 2-well glass culture slides (3 wells per condition; Lab-Tek, ThermoFisher, Carlsbad, CA USA) under the same conditions used above. When the incubation was concluded, the slides were rinsed three times in 1x PBS, fixed for 15 min in 4% PFA and stored covered in 1x PBS at 4 °C until analysis. Cells were imaged using an EVOS FL Auto Cell Imaging System (ThermoFisher, Carlsbad, CA USA) and analyzed using either the EVOS software or ImageJ (NIH, Bethesda, MD USA). Variables analyzed were nuclear counts normalized to protein content as measured by microBCA obtained for cells used for western immunoblotting, and cell area. Nuclei were visualized by staining with DAPI and imaged by fluorescence microscopy. Nuclear counts were determined by stitching together a 3×3 grid of 10x images and averaging the nuclear count of each stitched image then expressed as nuclei per nanogram protein. Average cell area was calculated by tracing 300 cells per condition (100 per well) using a brightfield image obtained at 20x magnification using the trace tool in ImageJ.

### 2.3 RNA Isolation and RT-PCR

Cells were lysed with 700 µL of Qiazol (Qiagen, Valencia, CA USA), placed into 1.5 ml microcentrifuge tubes and homogenized by vigorous vortexing. Chloroform (140 µL) was added and the solution was phase-separated by centrifugation at 14,000*g* for 15 min at 4 °C. The clear supernatant fraction was collected and RNA, including miRNA, was isolated and collected with a column according to the manufacturer’s instructions (miRNeasy Mini Kit; Qiagen, Valencia, CA USA). RNA was quantified using a Nanodrop 1000 (ThermoScientific, Carlsbad, CA USA). All samples had 260/280 ratios over 1.9. 1 µg of RNA was mixed with an RT primers pool (Applied Biosystems) for all of the miRNAs for the current study and reverse transcribed at 16 °C for 30 min, followed by a 30 min step at 42 °C and finished with a 5 min step at 85 °C (TaqMan miRNA Reverse Transcription Kit; Life Technologies, Carlsbad, CA USA). qPCR was conducted with gene expression assays for miR-1, miR-23a/b, miR-27b, miR-133a/b, miR-145, miR-206 and miR-486 using specific TaqMan miR assays (Applied Biosystems, Carlsbad, CA USA). The default qPCR protocol was used, specifically, a 10 min holding step at 95 °C, then 40 cycles of a 15 s step at 95 °C followed by a 60 s step at 60 °C. Individual ΔΔCts were calculated by using the equation:

(Experimental sample Gene of Interest - Average 1 D Vehicle Gene of Interest) – (Experimental sample Housekeeping Gene – Average 1 D Vehicle Housekeeping Gene).

U6 RNA was used as the housekeeping gene. At the 1 and 3 d timepoints, respectively, the U6 Cts for the experimental groups (mean +/- SD) were as follows: Con=21.87+/- 0.15 and 21.73+/- 0.17; 100 ng/ml myostatin=21.62+/- 0.21 and 21.79+/- 0.12, 100 ng/ml myostatin + SB431542=21.77+/- 0.18 and 21.87+/- 0.13; 1 µg/ml myostatin=21.84+/- 0.19 and 21.66+/- 0.14; 1 µg/ml + SB431542=21.68+/- 0.14 and 21.73+/- 0.13; SB431542=21.84+/- 0.06 and 21.81+/- 0.19. Fold-change for each sample was calculated as 2 raised to the power of the -ΔΔCt. [28].

### 2.4 SDS-PAGE and Western Immunoblotting

Cells were lysed with 1x RIPA buffer supplemented with protease and phosphatase inhibitors. The supernatant was collected and analyzed for protein content with a microBCA kit (Pierce, Carlsbad, CA USA). Lysates (30 µg of protein) were mixed 1:1 in Laemlli sample buffer and boiled for 3 min. Protein was then separated using a 10% polyacrylamide gel and transferred to a PVDF membrane. The membrane was stained with a Ponceau S solution to visualize protein loading and destained with 0.1 M NaOH. Membranes were quickly rinsed in Tris-buffered saline with 0.05% Tween 20 (TBS-T) and placed in a blocking solution of 5% bovine serum albumin (BSA) in TBS-T for an hour and incubated at 4 °C overnight with the primary antibody in 1% BSA/TBS-T solution. An antibody that detected both phosphorylated serine(465/467) Smad2/serine(423/425) Smad3 (#8828) and total GAPDH (#5174; Cell Signaling, Beverly, MA USA) were used in a 1:1000 dilution while MyoD (ab64159; Abcam, Cambridge, MA USA) was used at 1:500. After the overnight incubation, membranes were rinsed in TBS-T and placed in a 1:2000 solution of a horseradish peroxidase-conjugated secondary antibody (Cell Signaling, Beverly, MA USA) and 1% milk and TBS-T for an hour at room temperature. Membranes were rinsed in TBS-T and incubated with a horseradish peroxidase chemiluminescent substrate (ECL Prime, GE Healthcare; Marlborough, MA USA) for 5 min and imaged using a digital imager. The bands were quantitated with ImageQuant software (GE Healthcare; Marlborough, MA USA) and normalized to total GAPDH.

### 2.5 Statistics

Due to the limited effects of 100 ng/ml of myostatin on miRNA expression, an additional 3 wells was studied to confirm that this dose was largely ineffective. Thus, n=6 for gene expression studies for the vehicle and 100 ng/ml of myostatin groups and n=3 for all other experiments was used. All statistical analyses were completed using Prism 7.0 statistical software (Graphpad, San Diego, CA USA). Two-way ANOVAs (Treatment × Time) were used to detect interaction effects at p<0.05 for gene expression and morphology. Follow-up analyses for simple means differences were tested with one-way ANOVAs and Sidak’s multiple comparisons tests. The effect of myostatin and SB431542 on protein expression, as well as significant main effects, were analyzed with one-way ANOVAs at each time point with Sidak’s multiple comparisons tests when appropriate. Significance was noted when p<0.05.

## 3 Results

### 3.1 Cell morphology and size

**Fig. 1:**
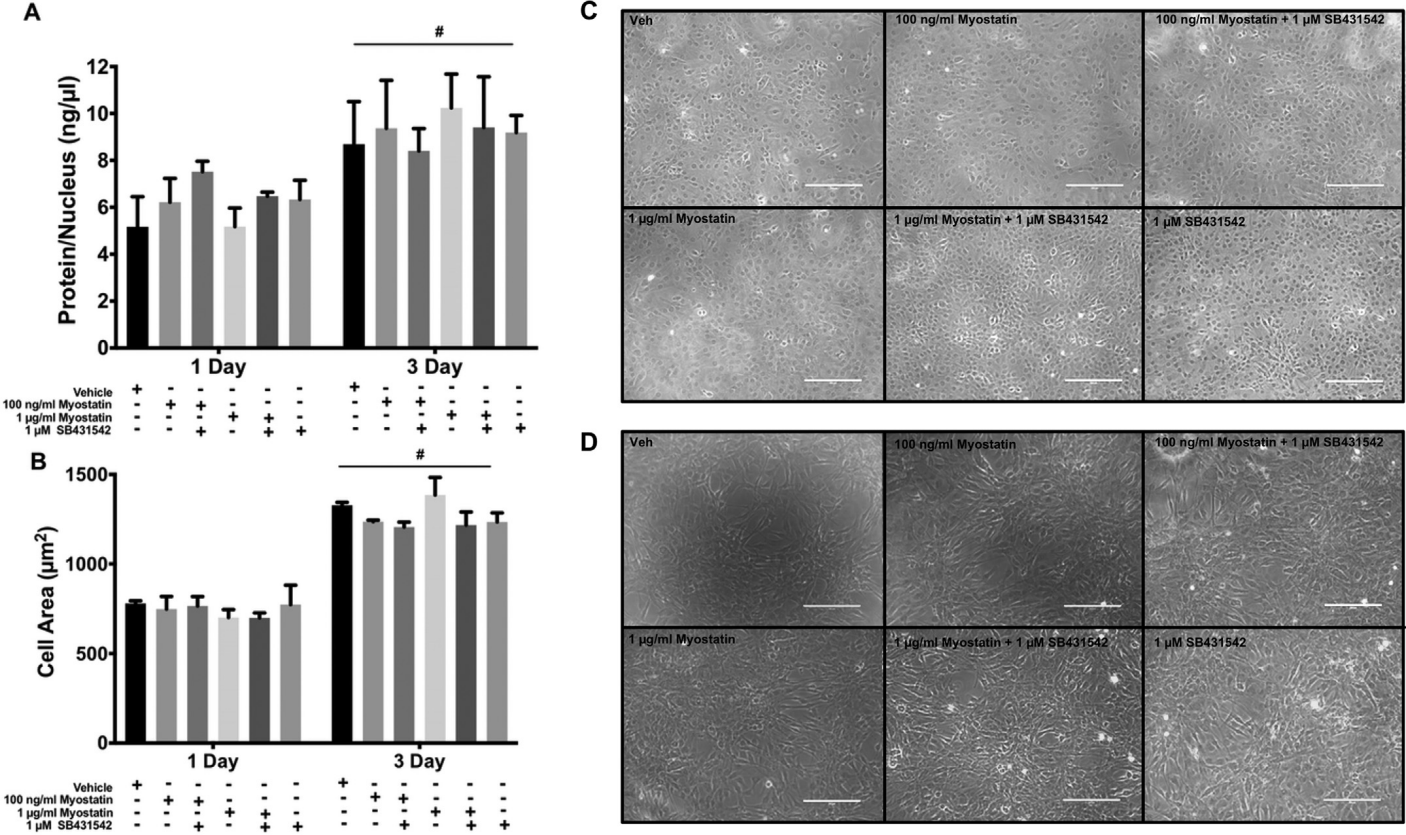
Morphological change is related to time but not myostatin or ALK4/5/7 inhibition in differentiating myoblasts. A) Protein content per nucleus and B) cell area. Representative photomicrographs can be seen for each treatment for cells at C) 1 D and D) 3 D. Significance was set at p<0.05. (#) represents main effect of time at p<0.0001. Data are presented means±SEM and n=3 wells per group.

Treatment of cells with myostatin or pharmaceutical inhibitors of Smad activation may alter cell size and morphology as well as myoblast differentiation. This possibility was evaluated by examining cell size, protein content, and morphology. Examination of cells by brightfield microscopy revealed that fusion of myoblasts had not occurred under any of the conditions tested and that morphology was similar for cells treated with vehicle, myostatin or SB431543 (Fig. 1C-D). However, we confirmed that our cell line does fuse and form myotubes. Brightfield images showed that at day 5 post-induction of differentiation, there were numerous myotubes present (Supplemental Fig. 1A) and by day 7 (Supplemental Fig. 1B), myotubes were more developed, thus confirming that this model is appropriate for investigating the role of myostatin in differentiating myoblasts. To determine whether the treatments altered cell size, we investigated whether the treatments changed the protein content per nucleus or cell area. There were no differences in protein content per nucleus between treatments at 1 or 3 D. However, we observed greater protein content per nucleus at 3 D compared to 1 D (Fig. 1A) indicating growth of the cells. Quantitation of the area of single cells revealed similar findings. There was not a significant difference in cell size between treatment groups at either 1 or 3 D. Cell size was greater at 3 D versus 1 D (Fig. 1B).

### 3.2 Changes in MyoD and pSmad2/3 at 1 and 3 days post-induction of differentiation

**Fig. 2:**
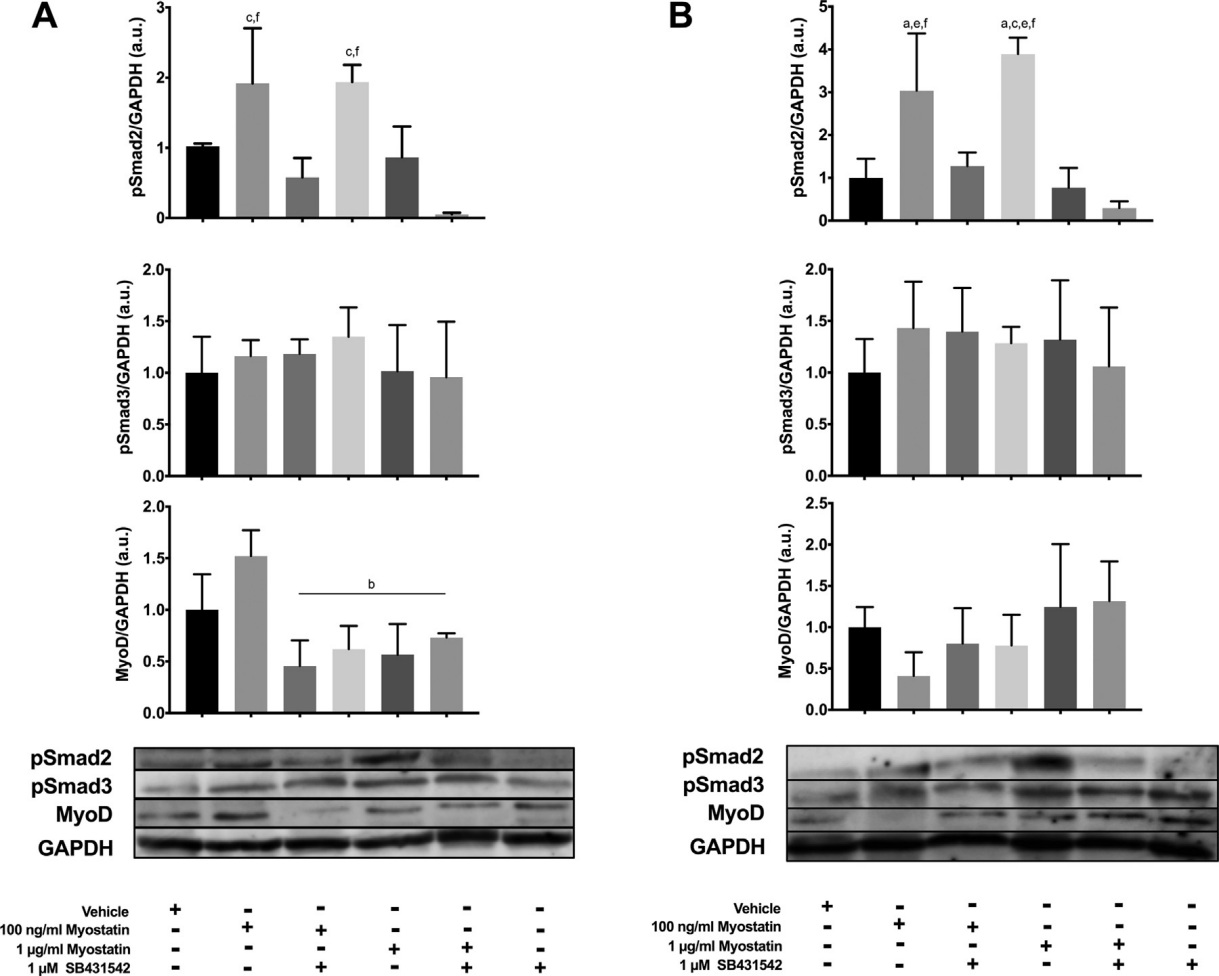
Myostatin and SB431542 alters Smad2 but not Smad3 phosphorylation after 1 and 3 d in differentiating media, with minimal differences in MyoD expression. Panels show: A) 1 D bar graphs and representative blots and B) 3 D bar graphs and representative blots. Protein levels were normalized to GAPDH and representative images slightly adjusted with a gamma correction. Statistically significant differences are noted as b=compared to 100 ng/ml myostatin, c=compared to 100 ng/ml myostatin + SB431542, f=compared to SB431542 alone. Data are presented means±SEM and n=3 wells per group.

To confirm that myostatin and SB431542 altered Smad activation under the conditions of the experiments, as has been previously reported in the literature, [19] levels of phosphorylated and total Smad2 and Smad3 were analyzed by Western blotting. C2C12 cells were induced to differentiate and left for 1 or 3 d; media was supplemented with 100 ng/ml or 1 µg/ml of myostatin with or without 1 µM SB431542, or 1 µM SB431542 alone, for the final 24 h before harvesting the cells. At 1 and 3 d post-induction of differentiation, incubation with myostatin increased pSmad2 levels by 2-fold compared to cells treated with SB431542 and 3-fold compared to controls (Fig. 2A and B) whereas SB431542 reduced pSmad2 levels below those present in controls, confirming the expected effects on Smad2 signaling. Levels of pSmad3 were not altered under the conditions of the experiment (Fig. 2A and B), consistent with prior reports. [19] At 1 d post-induction of differentiation, MyoD levels were reduced in cells treated with SB431542, high levels of myostatin or both (Fig. 2A). MyoD expression was not significantly altered across treatments at the 3 D time point (Fig. 2B). These results confirm that both myostatin and SB431542 altered MyoD levels, and presumably cell differentiation, when added during the first day after initiating myogenic differentiation whereas these agents had no effect at later times during differentiation. The fact that both myostatin and SB431542 reduced MyoD at 1 D argues against the reduction in MyoD being a direct effect of myostatin and in favor of indirect disturbances of the regulatory mechanisms responsible for MyoD expression and protein stability.

### 3.3 miRNA expression

#### 3.3.1 myomiRs

**Fig. 3:**
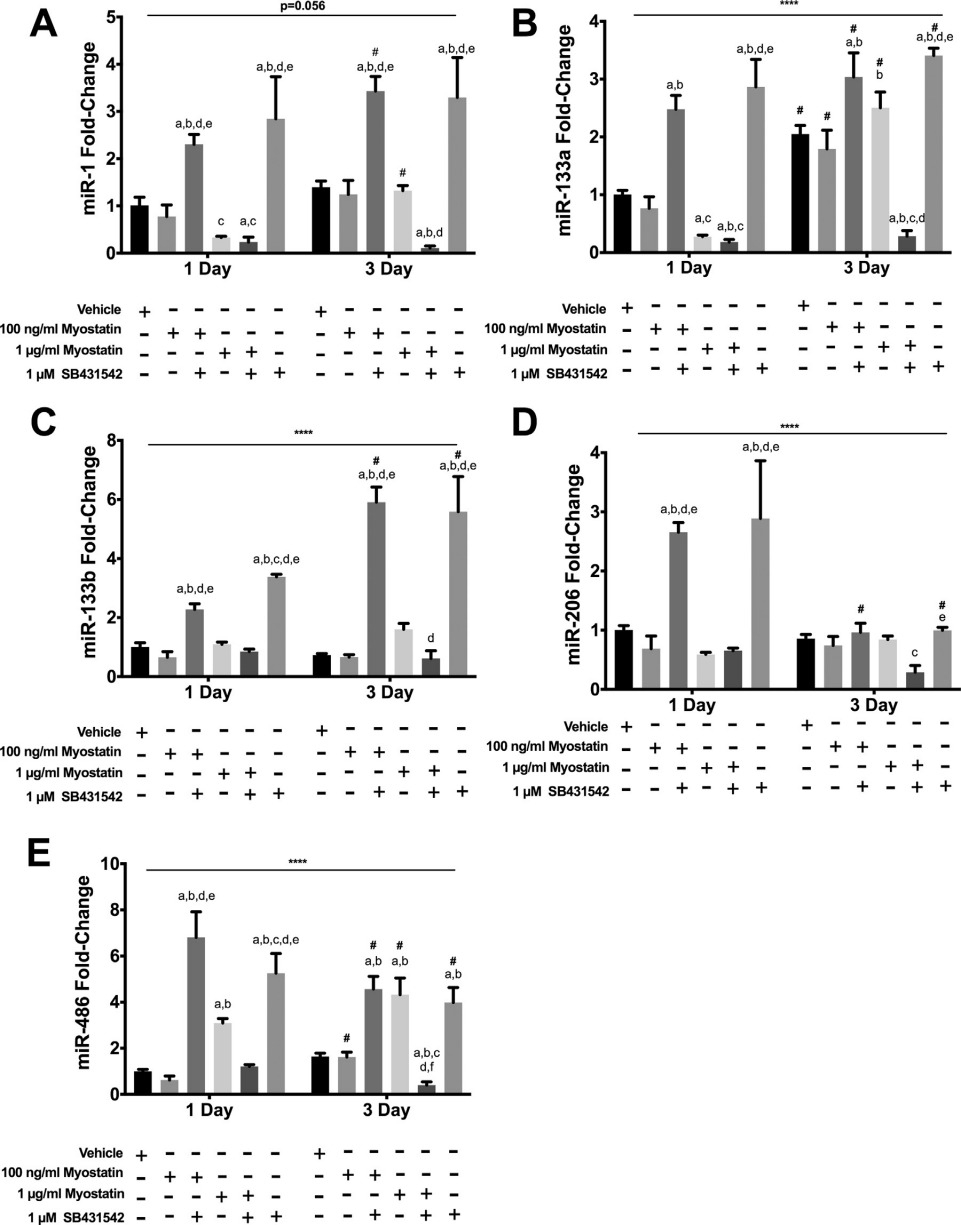
Effects of myostatin and/or SB4315342 on myomiR expression. C2C12 cells were incubated in differentiation media for 1 or 3 d. Myostatin, SB431542 or both were added to media 24 h before harvesting cells for analysis. Panels show transcript levels determined by RT-qPCR for: A) miR-1; B) miR-133a; C) miR-133b; D) miR-206; E) miR-486. (****) denotes an interaction at P<0.0001. Significant differences are noted as a=compared to Vehicle, b=compared by 100 ng/ml myostatin, c=compared to 100 ng/ml myostatin + SB431542, d=compared to 1 µg/ml myostatin and e=compared to 1 µg myostatin + SB431542 while #=compared with same treatment at 1 D time point. Data are presented means±SEM and n=6 wells for vehicle and 100 ng/ml of myostatin and n=3 for all other groups.

In low serum conditions, C2C12 myoblasts exit the cell cycle and begin to differentiate. There were significant treatment × time interaction effects in regards to the expression of the canonical myomiRs as well as the muscle-enriched miR-486 (Fig. 3). Overall, effects of myostatin and/or SB431542 on myomiR expression were not related to changes of MyoD protein levels. At 100 ng/ml, myostatin did not alter myomiR expression (Fig. 3). At 1 µg/ml myostatin reduced expression of miR 1 (Fig. 3A) and miR-133a (Fig. 3B), did not alter that of miR-133b (Fig. 3C) or miR-206 (Fig. 3D), and resulted in greater expression of miR-486 (Fig. 3E). At 3 D, myostatin did not alter myomiR expression (Fig. 3). The ALK4/5/7 inhibitor SB431542 increased expression of miRs 1, 133a, 133b, 206 and 486 at 1 D when administered alone or in combination with low but not high dose myostatin (Fig. 3). At 3 D, the inhibitor increased expression of miR 1, 133a, 133b and 486 but did not alter miR-206 expression (Fig. 3). Unexpectedly, when combined with the inhibitor, 1 µg/ml of myostatin reduced expression of the myomiRs and miR-486 at 3 D compared to untreated cells (Fig. 3).

#### 3.3.2 miRs 23a/b, 27b and 145

**Fig. 4:**
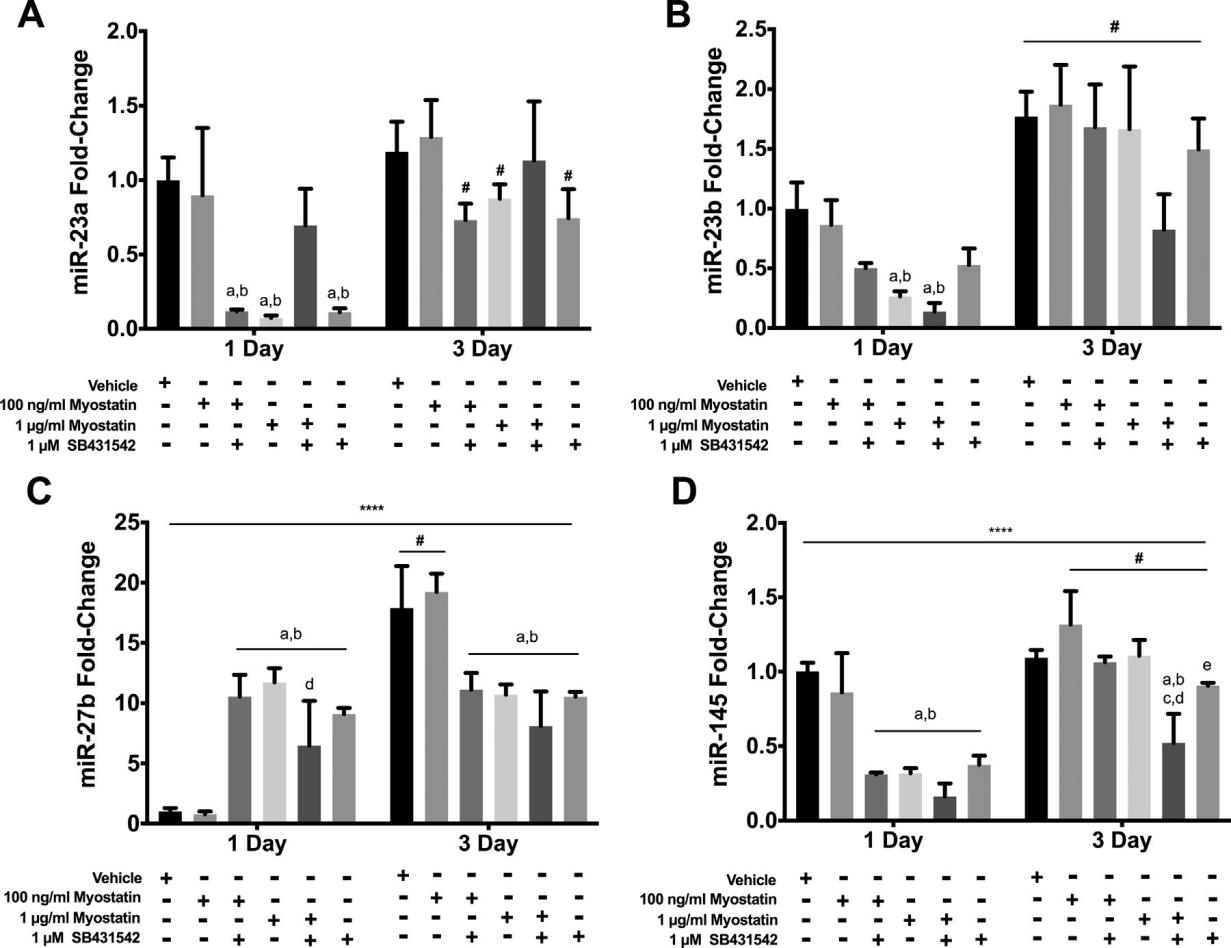
Effects of myostatin and/or SB4315342 on expression of other highly expressed miR. Myostatin, SB431542 or both were added to media 24 h before harvesting cells for analysis. Panels show transcript levels determined by RT-qPCR for: A) miR-23a; B) miR-23b; C) miR-27b; D) miR-145. (****) denotes an interaction at P<0.0001. Significant differences are noted as a=compared to Vehicle, b=compared by 100 ng/ml myostatin, c=compared to 100 ng/ml myostatin + SB431542, d=compared to 1 µg/ml myostatin and e=compared to 1 µg myostatin + SB431542 while #=compared with same treatment at 1 D time point. Data are presented means±SEM and n=6 wells for vehicle and 100 ng/ml of myostatin and n=3 for all other groups.

Incubation with 100 ng/ml myostatin did not alter levels of miRs 23a/b, 27b or 145 at 1 or 3 D (Fig. 4A-D). At 1 µg/ml, myostatin reduced expression of miRs 23a, 23b and 145 at 1 D and of miRs 23a and 27b at 3 D. SB431542 reduced expression of miRs 23a and 145 at 1 and 3 D, and of miR-27b at 3 D (Fig. 4A-D). Effects of SB431542 alone or together with myostatin were similar to those of myostatin alone for miR-23a, 23b, 27b, and 145 (Fig. 4A-D). SB431542 raised expression of miR-145 (Fig. 4D). Notably, the reduction at 1 D of miR-23b and miR-145 observed with myostatin and/or SB431542 correlated with reduced MyoD expression as did the absence of significant effects of these agents on miR 23a, miR-145 and MyoD protein at 3 D. Expression of miR-27b was, interestingly, elevated at 1 D by myostatin and/or SB431542, when MyoD was lowered by these agents, and reduced by these drugs at 3 D, a time when these molecules did not alter MyoD.

## 4 Discussion

The above studies aimed to determine whether myostatin modulated expression of a selected set of miRNAs in differentiating C2C12 myoblasts. The miRNAs included three miRNAs that are downregulated in muscle after SCI and are known to target mRNA transcripts encoding proteins involved in signaling by myostatin and other TGFβ family members (miR-23a, miR-145 and miR-206) [15]. The experiments were conducted using differentiating C2C12 myoblasts. While neither myostatin nor SB431542 altered cell size, SB431542 and 1 µg/ml myostatin reduced MyoD expression at 1 D; thus, a potential effect of these treatments on cellular differentiation cannot be excluded despite the fact that cell size and morphology were unaltered. Of note, these molecules did not alter MyoD expression at 3 D. Because myostatin at 1 µg/ml and SB431542 both altered expression levels at 3 D, when MyoD levels were not altered, the data support the conclusion that myostatin and ALK4/5/7 signaling regulate expression levels of these transcripts in differentiating C2C12 cells.

The reduction of pSmad2 after adding SB431542 suggests that there is activation of signaling through Smad2/3 under basal conditions, possibly as a result of myostatin or other TGFβ family members in the horse serum added to the growth media. At one day after inducing differentiation, effects of myostatin on miRNA-expression were dose-related; the (100 ng/ml) dose of myostatin did not alter expression of any of the miRNAs examined whereas the higher dose (1 µg/ml) suppressed miR-1 and miR-133a at 1 but not 3 D, and also reduced miR-23a, miR-23b and miR-145. The repression of miRNA expression by myostatin at 1 D was at least partially blocked by the ALK4/5/7 inhibitor. In addition, at 1 D, the inhibitor virtually eliminated pSmad2 and elevated expression of miRs 1 and 23a/b. The time after initiating differentiation was a determinant of the response to myostatin or the ALK4/5/7 inhibitor suggesting that in some way, the cellular context (e.g., degree of differentiation) is important to myostatin and ALK/4/5/7 signaling and their effects on miRNA expression. Specifically, the pattern of the changes elicited by myostatin or the ALK4/5/7 inhibitor at 3 D were similar to those at 1 D for miR-1 and miR-133a and miR-486, though not identical, while changes in miR-27b expression induced by these treatments were completely different at 1 and 3 D. The above findings support the view that under some conditions at least, myostatin represses expression of at least two miRNAs that target expression of proteins involved in signaling downstream of myostatin: miR-23a and miR-145. One might propose therefore a feed-forward amplification of myostatin signaling via repression of the expression of these miRNAs.

There were, however, some discordant findings in our data, such as the finding that both myostatin and the ALK4/5/7 inhibitor elevated miR-486 expression, which cannot be explained by the action of myostatin signaling through ALK4/5 alone, and which presumably reflect the action of other TGFβ family members with distinct actions on miRNA expression that act through parallel pathways involving either ALK4/5 or ALK7.

Alterations in miRNA expression induced by myostatin and ALK4/5/7 may have other effects on muscle homeostasis as well. Transcription of miR-1, miR-133a/b and miR-206 generally follows similar patterns during muscle differentiation, in part because genes for these miRNAs are found in two clusters, the miR-1/133a cluster and the miR-206/133b cluster [29], [30]. It is therefore not surprising that effects of myostatin, SB431542, or myostatin in the presence of SB431542, on myomiR expression occurred in a coordinated fashion for these miRs. Overall, the changes in myomiR expression induced by myostatin would be expected to repress differentiation of myogenic precursors. miR-1 and miR-206 share a similar sequence and promote muscle differentiation by targeting proteins such as Pax7, HDAC4 and connexin43 [30], [31] while miR-133a/b maintain a proliferative state through repression of SRF [30]. However, there is evidence which suggests that miR-133a/133b may have a role in myoblast differentiation by targeting non-SRF mRNA. [31] Our data suggests that the miR-206/133b cluster is less sensitive to high levels of myostatin compared to the miR-1/133a cluster, but that both clusters appear to be regulated by ALK/Smad2/3 signaling.

miR-23a/b has been implicated in muscle atrophy because it targets two muscle-restricted E3 ligases, MuRF1 and MAFbx, which are involved in the breakdown of muscle proteins during atrophy induced by diverse conditions including denervation and glucocorticoid administration [32], [33]. The role of miR-145 in muscle is unknown although its expression is decreased following denervation [34] and it can target Cited2, a transcription factor which when overexpressed protects myotubes from glucocorticoid-induced atrophy [35]. There is also the chance that potential changes in the miRNA profile due to high levels of myostatin could have an effect beyond regulating transcription of intracellular mRNA targets. miRNAs can be packaged into exosomes and delivered to the circulatory system to act as biomarkers or potentially acting in an paracrine or endocrine manner to regulate important physiological systems such as metabolism. [36], [37].

Our findings regarding the myomiRs are largely in agreement with Rachagani et al. [38] who showed myostatin knockout mice have increased myomiR expression in the pectoralis muscle compared to partial knockdown and wild type mice. However, our data is not completely consistent with the literature with respect to miR-486. Specifically, a myostatin knockout led to increased expression of miR-486 in the mouse tibialis anterior and recombinant myostatin decreased mRNA expression of the *Ank1* gene, the gene from which miR-486 is derived [39].

miR-27b can target the 3′-UTR of Pax3, reduce Pax3 levels and drive the differentiation program of muscle cells [22], and miR-27b may be responsible for determining fiber type by targeting myostatin mRNA [23]. It is therefore interesting that at higher doses, myostatin regulated miR-27b expression. At 1 d post-induction of differentiation, when myostatin may repress initiation of differentiation, myostatin stimulated miR-27b expression suggesting that high levels of myostatin may drive miR-27b expression as a negative regulator of myostatin release. Conversely, at 3 d post-induction of differentiation, myostatin suppressed miR-27b expression which could be interpreted as another form of feed-forward amplification of myostatin signaling through allowing greater tissue myostatin release. These data reinforce the conclusion above that influence of myostatin and, more broadly, signaling downstream of ALK4/5/7, on expression of these miRNAs is highly dependent on the stage of differentiation of cells in the myogenic lineage.

The influence of myostatin and the ALK4/5/7 inhibitor on miRNAs expression has broader implications. miR-23a and miR-23b differ by only a single nucleotide and target two E3 ubiquitin ligases known to participate in skeletal muscle atrophy, MuRF1 and MAFbx [32]. Ectopic overexpression of miR-23a *in vitro* and *in vivo,* as well as miR-23a overexpression in transgenic mice, prevented dexamethasone-induced muscle atrophy [32]. Our data show that high levels of myostatin can greatly lower the expression of miR-23a/b and inhibition of ALK signaling in a similar reduction 1 D after differentiation, suggesting a novel pathway of myostatin action through modulating miR-23a/b expression levels. Studies *in vivo* or *ex vivo* would be anticipated to shed more light on the question of the influence of myostatin on expression of these miRNAs in skeletal muscle.

## Appendix B Supplementary material

Supplementary material **Supplemental Fig. 1** Brightfield microscopy showing formed myotubes at A) 5 days and B) 7 days after the induction of differentiation of C2C12 myoblasts. Reference bars =400 µm for the 10x field and 200 µm for the 20x field.
