# Supplementary figures and images for: Recombinant myostatin reduces highly expressed microRNAs in differentiating C2C12 cells
Source: Biochem Biophys Rep. 2017 Jan 17;9:273–80. doi: 10.1016/j.bbrep.2017.01.003 (PMC5500170; doi:10.1016/j.bbrep.2017.01.003)

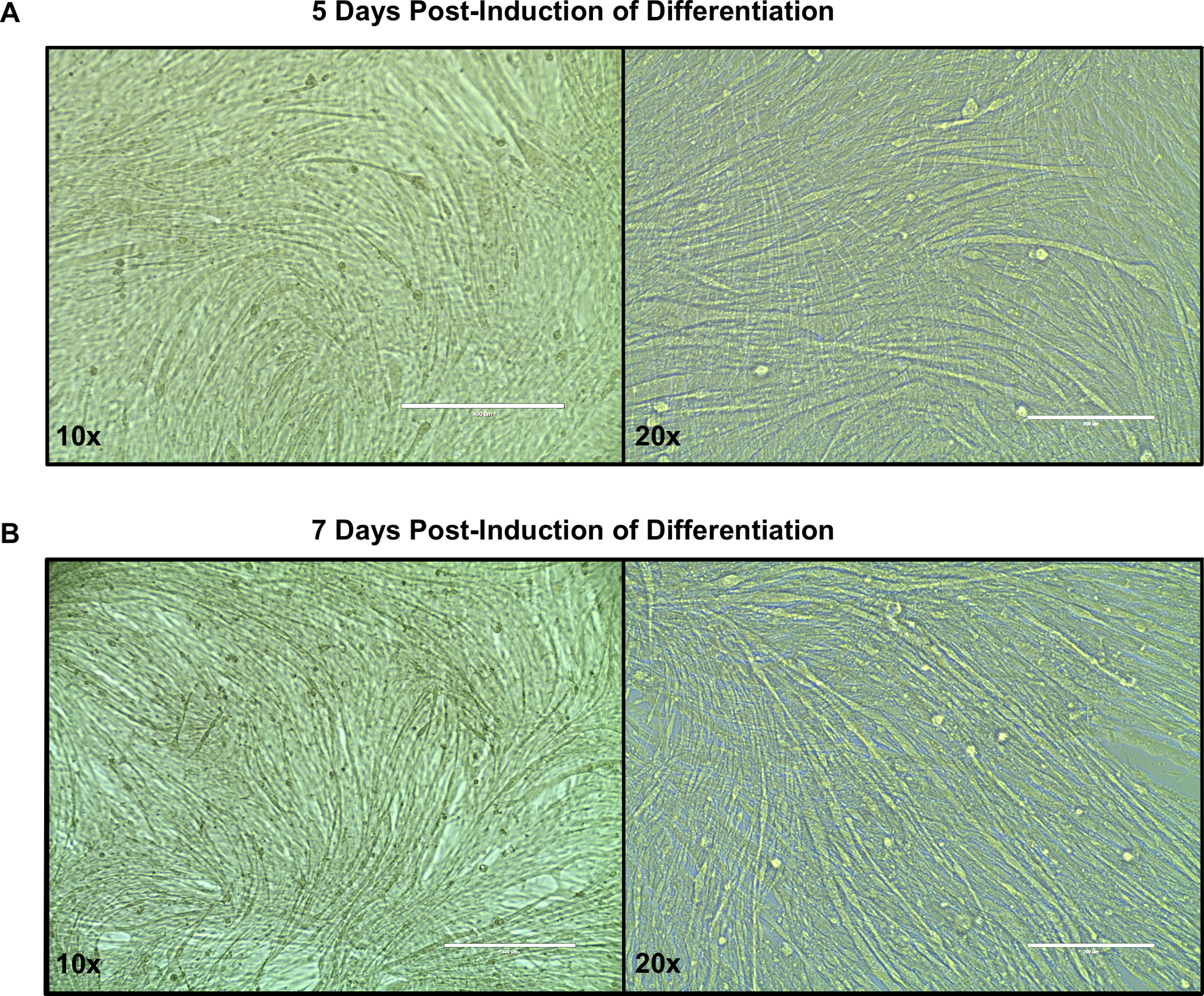

Supplement: Supplementary file 5 — Supplementary material Supplemental Fig. 1 Brightfield microscopy showing formed myotubes at A) 5 days and B) 7 days after the induction of differentiation of C2C12 myoblasts. Reference bars =400 µm for the 10x field and 200 µm for the 20x field. [file mmc5.zip › BBR R2 Suppemental Figure Not Compressed.tif]
